# Wheat–*Fusarium graminearum* Interactions Under *Sitobion avenae* Influence: From Nutrients and Hormone Signals

**DOI:** 10.3389/fnut.2021.703293

**Published:** 2021-09-08

**Authors:** Kun Luo, Thérèse Ouellet, Huiyan Zhao, Xiukang Wang, Zhensheng Kang

**Affiliations:** ^1^State Key Laboratory of Crop Stress Biology for Arid Areas, College of Plant Protection, Northwest A&F University, Xianyang, China; ^2^Shaanxi Key Laboratory of Chinese Jujube, College of Life Science, Yan'an University, Yan'an, China; ^3^Ottawa Research and Development Centre, Agriculture and Agri-Food Canada, Ottawa, ON, Canada

**Keywords:** pathogenicity, mycotoxin production, defense responses, phytohormones crosstalks, auxin biosynthesis

## Abstract

The English grain aphid *Sitobion avenae* and phytopathogen *Fusarium graminearum* are wheat spike colonizers. “Synergistic” effects of the coexistence of *S. avenae* and *F. graminearum* on the wheat spikes have been shown in agroecosystems. To develop genetic resistance in diverse wheat cultivars, an important question is how to discover wheat–*F. graminearum* interactions under *S. avenae* influence. In recent decades, extensive studies have typically focused on the unraveling of more details on the relationship between wheat-aphids and wheat-pathogens that has greatly contributed to the understanding of these tripartite interactions at the ecological level. Based on the scientific production available, the working hypotheses were synthesized from the aspects of environmental nutrients, auxin production, hormone signals, and their potential roles related to the tripartite interaction *S. avenae*–wheat–*F. graminearum*. In addition, this review highlights the relevance of preexposure to the herbivore *S. avenae* to trigger the accumulation of mycotoxins, which stimulates the infection process of *F. graminearum* and epidemic of Fusarium head blight (FHB) in the agroecosystems.

## Introduction

Common wheat (*Triticum aestivum* L.) is one of the most cultivated cereals worldwide, occupying ~240 million ha worldwide and feeding ~40% of the world population (1). However, the production of wheat is severely threatened by many intruders. In the past years, the main areas of wheat plants infestation by cereal aphids and Fusarium head blight (FHB) have gradually overlapped in the wheat-producing regions of China (Chinese National Agro-Tech Extension and Service Center, accessed 2015–2021). Currently, several economically important aphid species severely threaten the wheat production in China, such as the English grain aphid *Sitobion avenae* Fabricius, greenbug, *Schizaphis graminum* Rondani, and bird cherry-oat aphid *Rhopalosiphum padi* L. (Hemiptera: Aphididae), while the fungus *Fusarium graminearum* Schwabe and the Asian species *F. asiaticum* (Hypocreales: Nectriaceae) are the primary pathogens causing FHB in China. Among these species, the phloem feeders *S. avenae* and *F. graminearum* are the two economically important pests residing on the wheat spikes (2, 3). The aphid *S. avenae* penetrates the phloem cell and sucks the phloem sap with their stylet-like mouthpart, frequently reduces the wheat grain yield by as much as 30%, sometimes by more than 60% (2). *F. graminearum* has the capacity to destroy potentially high-yielding crops within a few weeks prior to harvest (4). In addition, close to the wheat flowering stage, it is common for *S. avenae* and *F. graminearum* to sequentially coexist on the same plants in agroecosystems. When *S. avenae* infests the wheat spikes earlier than *F. graminearum*, it has been demonstrated to accelerate FHB progression and deoxynivalenol (DON) accumulation (5, 6). In addition, the longer the period of preinfestation by *S. avenae* prior to the infection with *F. graminearum*, the greater the amount of pathogen DNA that was accumulated (6), suggesting a “commensal” relationship between the two species and the coexistence of *S. avenae* and *F. graminearum* on the wheat spikes, resulting in devastating damage to the grain yield and quality and severely threatening the food safety of China. Apart from the negative effect on the grain yield, the accumulation of mycotoxins caused by *F. graminearum* also seriously reduces the quality of the grain (7). *F. graminearum* produces trichothecene (TRI) mycotoxins, such as nivalenol (NIV) and DON, and its acetylated derivatives. The toxin-contaminated wheat grains are often unsuitable agricultural products for human food or animal feed inflicting tremendous economic impacts on wheat production (8).

Although breeding wheat cultivars with aphid or Fusarium resistance is considered as an ideal measure to combat their attacks, resistant cultivars available in agricultural production remain relatively rare. Currently, insecticidal sprays are still crucial measures for controlling biotic damage, especially cereal pests used in agricultural production in the most wheat-producing regions in China (Chinese National Agro-Tech Extension and Service Center, accessed Apr. 2021). The insecticidal sprays could effectively control the damage from different biotic stresses in a short time; however, economically irrational insecticidal sprays in farming have led to the development of resistance to many insecticidal compounds in the pests and increased production costs. Therefore, a better understanding of the biological and ecological reasons why cereal aphid preinfestation positively affects the infection by *F. graminearum* will be an important issue for guaranteeing the food safety of China, even the food safety worldwide. To date, genetic and biochemical evidence from the previous studies, using aphids and/or *F. graminearum* or closely related fungal species has greatly contributed to a comprehensive view of this tripartite interaction. Therefore, this review focused on the recent progress in understanding the interaction between *F. graminearum* and its hosts preinfested with *S. avenae*, from the aspects of the nutrients and hormone signals.

## Aphids and Their Endosymbionts as Drivers of Reallocation of Plant Nutrients Involved in Wheat–*F. graminearum* Interactions

Carbon fixation *via* photosynthesis is a crucial process for plants to convert the sunlight energy into more complex photoassimilates. During the grain-filling stage, large amounts of photoassimilates are transported into the endosperm, contributing to the grain yield. Such photoassimilates present in phloem sieve elements constitute basic nutrients indispensable to *S. avenae*, providing carbon skeletons for the synthesis of more complex compounds and an energy source. To continuously feed on the nutrients from the host, *S. avenae* must overcome the sugar and nitrogen barriers to phloem sap utilization (9). The sugar barrier is caused by the high concentration of sugar in phloem sap, up to and often exceeding 1 M (mol·L^−1^) sugar, and a resultant osmotic pressure several times greater than the osmotic pressure of the body fluids of aphids (9, 10). In plants, sucrose is the major phloem-translocated component of photoassimilates and is transported from the synthesizing organs to sink organs by sieve tubes (11). To sustain the continuous flow of sucrose at high osmotic pressure into their gut, *S. avenae* must transform most of the assimilated sucrose into honeydew to reduce the osmotic pressure of the gut contents (10, 12). In contrast, the nitrogen barrier present in the phloem sap is caused by an unbalanced composition of free amino acids, containing only low concentrations of most of the essential amino acids that herbivores cannot synthesize (9). In order to compensate for the deficiency in essential amino acids of phloem sap, nitrogen source reallocation has been observed in the hosts previously infested with different species of aphids, such as *S. graminum* (Rondani) or Russian wheat aphid *Diuraphis noxia* (Mordvilko) (Hemiptera: Aphididae) (13, 14). In addition, most aphids are hosts to endosymbionts, with *Buchnera aphidicola* being an obligate species. *B. aphidicola* can synthesize the missing essential amino acids with a high concentration of non-essential amino acids and provide a nitrogen source not only for aphid growth and fecundity but also for honeydew secretions (9, 15). During feeding, *S. avenae* excretes large amounts of honeydew, which is primarily composed of sucrose and monosaccharides, with a limited amount of nitrogen compounds, such as amino acids, that are partly contributed by its endosymbiont (16, 17). Therefore, honeydew at the interface of plants is the primary and predominant exogenous carbohydrate and nitrogen source in many ecosystems and would play a dominant role in the tripartite interaction of *S. avenae*–cereal–*F. graminearum* (18).

In the wheat–*F. graminearum* interaction, the fungus directly removes the sucrose from the primary metabolism of the host for its own growth and the production of virulence factors (19, 20). The TRI mycotoxin DON produced by *F. graminearum* acts as a virulence factor and is essential for symptom development after initial wheat infection (21). This study aimed to reduce the massive yield losses and serious health concerns caused by FHB and focused on understanding the biosynthesis and regulation of the mycotoxin DON and its derivatives produced by *F. graminearum* (21, 22). In recent decades, considerable progress has been made in determining the genes responsible for DON biosynthesis in *F. graminearum* (23). Many reports have indicated that sucrose is an important inducer of the trichothecene gene (*TRI*) cluster required for the biosynthesis of DON (21). In particular, the genes *TRI5* and *TRI4* that catalyze steps in the biosynthesis of DON, are strongly upregulated in the liquid culture by sucrose, followed by a large increase in the accumulation of DON and derived compounds (24). Wheat spike tissues after anthesis are rich in carbon sources, such as sucrose and fructo-oligosaccharides. During the infection of wheat head tissues, both the plant and pathogenic invertases are upregulated, leading to the conversion of sucrose into monosaccharides, which can potentially disturb the source-sink balance and the partitioning of carbon sources in the plant tissues to promote TRI production. When wheat plants were pre-exposed to *S. avenae*, the presence of sucrose-rich honeydew from *S. avenae* appeared to provide abundant, ready-to-use carbon nutrients for *F. graminearum*, which in turn would be stimulated to produce increased amounts of mycotoxin DON (5).

Taken together, the above findings have led to the hypothesis that the coexistence of *S. avenae* and *F. graminearum* is helpful in increasing the levels and compositions of nutrients in wheat tissues, benefiting both aphids and fungi, potentially contributing to the development of epidemic conditions for FHB, and the accumulation of TRI mycotoxins.

## Defense Mechanisms Mediated by Wheat–*F. graminearum* Interactions Under *S. avenae* Influence

In response to the biotic attacks, plants coevolved a range of defense mechanisms, such as morphological, biochemical, and molecular mechanisms, to control the damage caused by immediate and subsequent colonizers. These plant defense mechanisms can be broadly categorized into constitutive and induced defenses (25). The constitutive defenses constitute the first physical barriers and are expressed as antixenotic, where certain characteristics of a plant, such as leaf surface wax, trichomes, and cell walls, make it less attractive to herbivores, and antibiosis, where the plant produces toxins, such as plant phenolics, flavonoids, tannins, dimboa, plant lectins, and proteinase inhibitors (25). These compounds can be either constitutively stored as inactive forms or induced in response to the insect or pathogen attack (25).

Once herbivores successfully colonize and begin feeding, plants trigger the induced defenses to counter the effects of herbivore attack. Over the past few decades, considerable progress has been made in understanding the induced responses in plants against different biotic attacks, and it has become an important topic in evolutionary biology and ecology (25, 26). The induced defenses are mostly mediated through the release of phytohormones that specifically activate the hormone-dependent response pathways against various attackers. The signaling molecules jasmonic acid (JA) and salicylic acid (SA) are recognized as major defense hormones (27–29). The extensive accumulation of JA and SA in plant tissues associated with the piercing sucking due to aphid feeding, resulting from the increased expression of enzymes in the JA and SA biosynthetic pathways, activates JA-/SA-mediated defense responses (27–30). In addition, *F. graminearum* is a hemibiotroph with both a biotrophic and a necrotrophic phase during the colonization of wheat plants. To counter the *F. graminearum* infection, the plant defense mechanisms associated with SA- and JA/ethylene (ET)-dependent defense can also be activated (31). *Arabidopsis* has shown that the coordinated and ordered expression of SA- and JA/ET-dependent defense responses were crucial to halt the *F. graminearum* infection (32). The experimental data provided by *Arabidopsis* mutants with defects in various defense-related signaling pathways suggested that the SA-dependent defense is generally accepted to be most efficient against the biotrophic pathogens, while JA- and ET-dependent defense is especially activated during the plant defense against necrotrophic pathogens (33–35). ET signaling is known mostly for its synergistic interaction with the JA pathway, probably because the expression of JA response genes is concomitantly activated with the components of ET signaling (36, 37). Gene expression data in other plant species, such as sorghum and wheat, have suggested that SA is the most important phytohormone in the plant defense against aphid herbivores and biotrophic pathogens (28, 38, 39). Together, SA signaling contributes to the understanding of the crucial roles of plant hormones in tripartite *S. avenae*–wheat–*F. graminearum* interactions, as discussed below.

**Figure 1:**
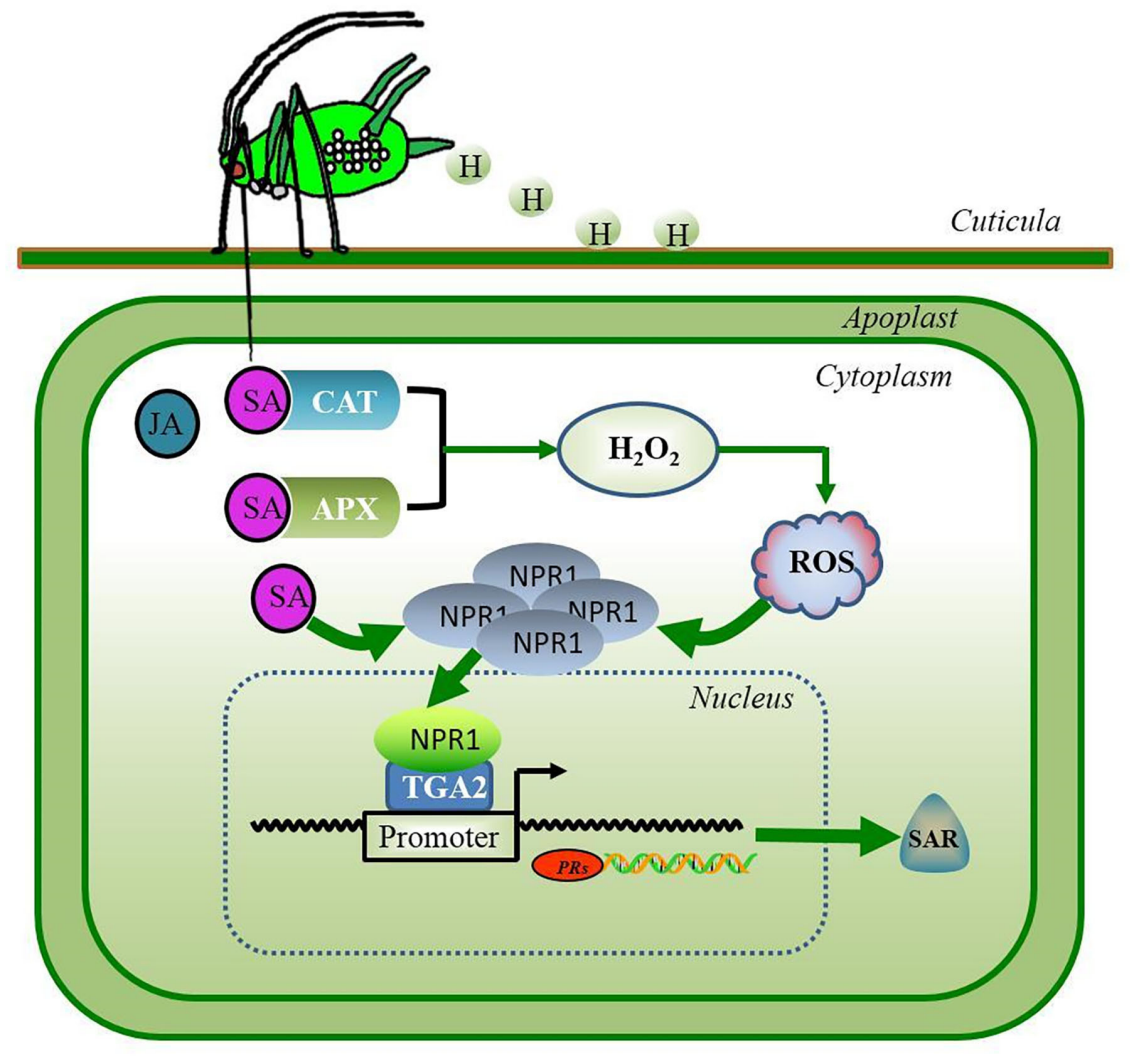
The feeding behavior of the piercing-sucking herbivore *S. avenae* induces hormone-mediated defense signaling in wheat. Salicylic acid (SA) is the primary phytohormone in plant defense against the aphid insects. The SA-binding proteins APX and CAT rapidly bind SA, resulting in the extensive accumulation of H_2_O_2_. Extra H_2_O_2_ rapidly disrupts ROS homeostasis that may induce the dissociation of oligomeric NPR1 into a monomeric form. The translocation of monomeric NPR1 into the nucleus leads to association with the transcription factor TGA2 in an enhanceosome, thereby activating the expression of defense genes and triggering SAR in plants. Green lines and arrows indicate the pathways of SA signaling. APX, ascorbate peroxidase; CAT, catalase; H, aphid honeydew; H_2_O_2_, hydrogen peroxide; JA, jasmonic acid; NPR1, non-expressor of pathogenicity-related genes 1; PRs, pathogenicity-related proteins; ROS, reactive oxygen species; SAR, systemic acquired resistance; TGA2, transcript factor TGACG binding II.

The generated SA can be perceived by SA-binding proteins (SABPs); catalase (CAT) was the first identified plant protein found to physically bind the SA using isotope ^14^C tagging detection (40–42). For decades, more researchers have identified additional SA receptors, such as ascorbate peroxidase (APX) (43–45). In plants, CATs and APXs are known to scavenge H_2_O_2_ generated during normal metabolism of oxygen and exposure to various biotic and abiotic stresses (43). The competitive binding of SA to CATs or APXs resulted in the inhibition of their scavenging activity toward H_2_O_2_ (41). The inhibition of CATs and APXs by SA binding was shown to lead to the accumulation of H_2_O_2_ in *Arabidopsis* plants, which then, activated the cellular redox changes and resulting in the dissociation of oligomeric non-expressor of PR genes 1 (NPR1) into its monomeric form (43, 46, 47). The translocation of monomeric NPR1 into the nucleus leads to the recruitment of the transcription factor TGA2 (TGACG binding II), and monomeric NPR1 for assembly into enhanceosome binds to the PR-1 promoter region, activates the expression of the SA-dependent pathogenicity-related protein 1 (PR-1) gene, and triggers the systemic acquired resistance (46–48) (Figure 1). In wheat, overexpression of the *Arabidopsis* NPR1 gene renders the plants resistant to FHB (49).

To better establish a parasitic relationship, the SA induced by the herbivore preinfestation has become a target of pathogens in many pathosystems in an attempt to reduce the host defenses. *F. graminearum* has been shown to metabolize SA as a source of carbon (50). The salicylate hydroxylase gene of *F. graminearum* (*FGSG_08116, FgNahG*), involved in an essential initial catabolic step converting SA into catechol, is widely distributed in hyphae; infections with knockout mutants Δ*FgNahG* showed reduced disease symptoms and fungal biomass in the spikes when compared to control (51). Another pathogenic *Fusarium* species, *Fusarium oxysporum*, can produce bioactive forms of JA through an iron 13S-lipoxygenase (FoxLOX) similar to the LOX enzymes utilized by plants for JA biosynthesis (52); the capacity of JA to stimulate a cascade of reactions in plants may allow the fungus to inhibit the expression of SA-dependent defense genes. If a similar ability to produce JA was shown for *F. graminearum*, a reduction in SA-dependent responses by the pathogenic fungus could improve the fitness of herbivores, such as *S. avenae* when they coexist on the same wheat plants.

When the production of H_2_O_2_ exceeds the amount that cells can gradually digest, the excess results in irreversible cellular cytotoxicity and oxidative damage to cell membranes. The extensive accumulation of H_2_O_2_ in the tripartite interaction may also elevate the expression of the DON biosynthesis machine in *F. graminearum*. For instance, the treatment of *F. graminearum* culture with exogenous H_2_O_2_ or with the fungicide prothioconazole, an inducer of H_2_O_2_, resulted in higher levels of expression of the *TRI4* and *TRI5* genes (53). High levels of DON were also observed in the wheat plants treated with prothioconazole (53). In addition, knockout mutants of the transcription factor Δ*FgAP1*, which are involved in the activation of transcription of antioxidant enzymes, showed upregulation of the TRI5 gene by ~5- and a 20-fold higher level of TRI production (54).

Advances in understanding the process of spike colonization by *F. graminearum* have revealed an important relationship between the accumulation of H_2_O_2_ and DON production (21, 55). When rain-splashing, wind-dispersal, or herbivore transfers conidium, or ascospores of *F. graminearum* to land on the exposed, vulnerable parts (glumae, floral cavity, lemma, palea, anthers, or punctures), or C- or N-rich parts of a wheat plant during or just after anthesis, the spores can germinate and penetrate into the plant (3, 21). At the advancing infection front, *F. graminearum* invades the wheat tissues using a biotrophic mode of nutrition. An initial superficial and intercellular spread of the fungal hyphae on the inner surface of plant tissues is followed by the living host tissue being surrounded by abundant intercellular, colonizing hyphae (56, 57). Thereafter, the infection hyphae form the infection cushions and foot-like structures, penetrating the host cells that lost their entire cellular content. In this phase, the rapid and transient generation of H_2_O_2_ in the damaged host plant tissues attacked by the herbivore and fungus often coincides with the hypersensitive response (HR)- and programmed cell death (PCD)-type responses; in general, these responses isolate subsequent biotroph colonizers and deprive them of nutrients required for further damage. To pursue its attack, *F. graminearum* switches its nutritional mode to necrotrophic, a more invasive intracellular growth, and survives off the dead host tissues (56, 57). During the necrotrophic phase, high DON concentrations are generated, triggering H_2_O_2_ synthesis, which results in the fungal growth and wheat cell death (21). Although *F. graminearum* inoculation could induce a greater expression of the genes associated with the cell wall reinforcement (5), the production of DON is thought to equip *F. graminearum* with a powerful capacity to impede the cell wall reinforcement processes in the infected spikes (58). Experiments with a Δ*TRI5* knockout mutant strain unable to synthesize DON confirmed that DON was required for the spread of *F. graminearum* in the rachis (21, 59). This evidence points to the DON production and the ability to thrive under oxidative stress conditions as efficient tools for *F. graminearum* to colonize and spread within the wheat host.

## Phytohormone Crosstalk Fine-Tunes Major Plant Defense Responses and Auxin Biosynthesis

One could expect that *S. avenae* and *F. graminearum* sequentially coexisting on the same plants would have stimulated a stronger upregulation of SA biosynthesis in wheat; however, the expression of PAL, part of the SA biosynthesis pathway, was significantly reduced in the wheat plants successively infested with *S. avenae* and then infected with *F. graminearum* when compared with the plants infected with *F. graminearum* alone (5). To counteract plant defense, pathogens have probably developed the ability to fine-tune the plant immune response to promote the disease (60). This strategy frequently involves antagonistic interactions between the signaling pathways for SA and JA/ET, where induction of one pathway always attenuates the other (33, 39, 60–63). Some regulators, such as MPK4 (mitogen-activated protein kinase 4), NPR1, transcription factors WRKY70, and TGA2, have been identified for their role in the crosstalk between SA- and JA-/ET- signaling pathways in the plant immunity (33, 39, 60–63). In addition, the interactions of growth-promoting phytohormones, such as auxin, cytokinin, abscisic acid (ABA), gibberellins, and brassinosteroid, shift the balance toward SA or JA/ET defense responses through either direct or indirect action, fine-tuning the plant immunity and constituting the network of phytohormone crosstalk (39, 64, 65). Using the pathosystem *Arabisopsis/Pseudomonas syringae* as a model, auxin was found to promote biotroph susceptibility associated with the JA/ET signaling pathways, while gibberellin was implicated in the biotroph resistance associated with the SA signaling pathway (39, 65). Other experiments showed that cytokinins also promoted biotroph resistance in an SA-dependent manner (66, 67), while brassinosteroids promoted biotroph resistance *via* an SA-independent pathway (68). The contribution of ABA is complex, as it has been associated with both biotroph and necrotroph susceptibility and implicated in the response to abiotic stress (39, 69). Moreover, many phytopathogens have the capacity to produce growth-promoting phytohormones or mimic molecules, rendering the host tissue more suitable for colonization, growth, and disease symptom development. Some pathogens have been shown to manipulate the host physiology by producing auxins and cytokinins, known regulators of plant growth and development, to accelerate the infection process (70, 71). For instance, the rice blast fungus *Magnaporthe oryzae* (Ascomycota) has been shown to synthesize cytokinins and auxin as a pivotal requirement for a successful infection (72, 73). *F. graminearum* does not have the capacity to produce cytokinins because its genome does not contain the required genes for cytokinin synthesis (74); however, we have recently shown that *F. graminearum* infection is associated with over 300-fold accumulation of auxin in the susceptible wheat head tissues, especially in the first few days of infection (75). Thus, the extensive increase in auxin accumulation in their hosts observed could be an important hallmark of pathogenicity for many pathogenic organisms.

**Figure 2:**
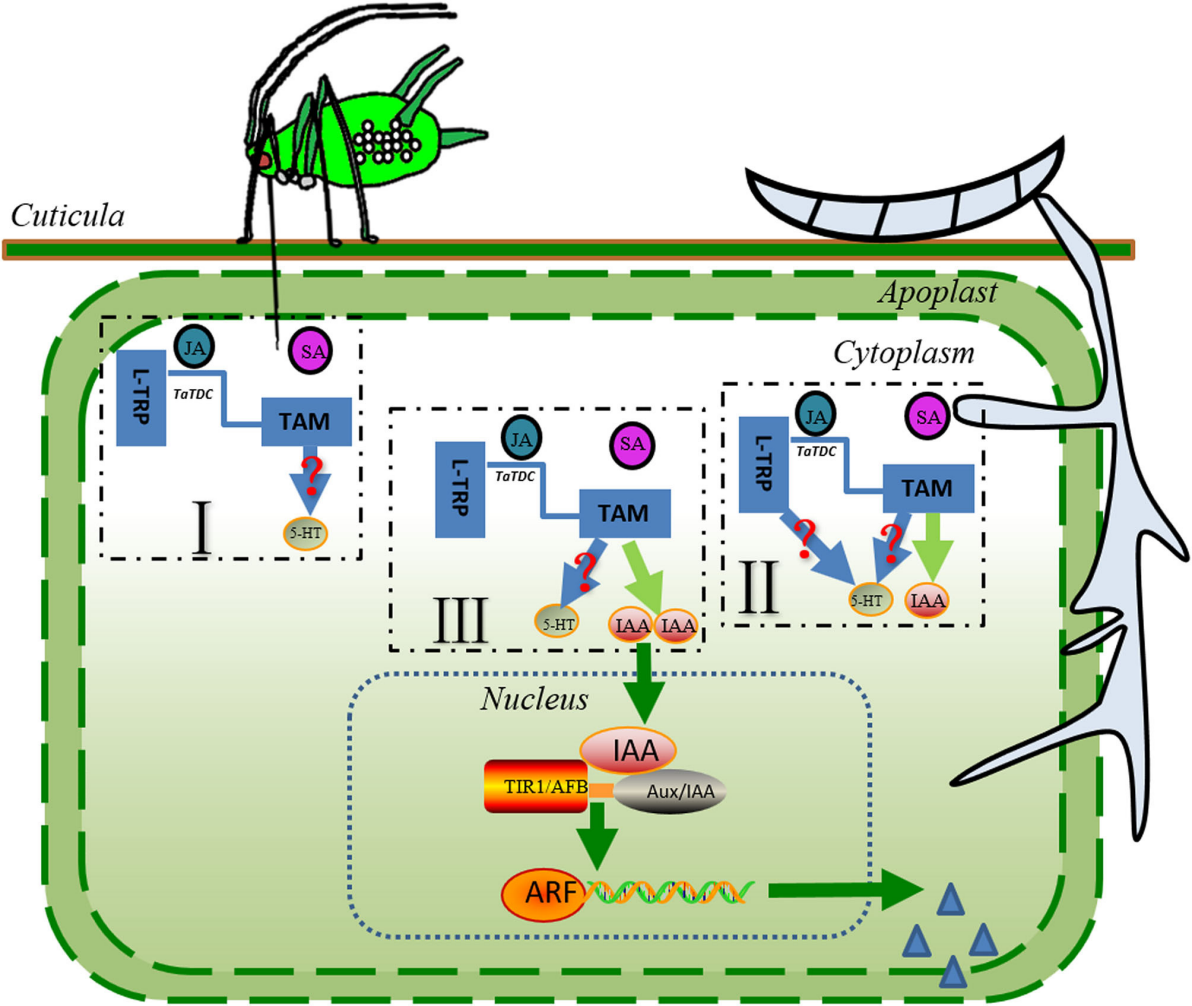
The potential pathways of L-TRP catabolism during sequential infestation with *S. avenae* and *F. graminearum*. The sequential colonization of *S. avenae* and *F. graminearum* induces the JA-mediated defense signaling, which may result in a higher level of expression of the *TaTDC* gene and the accumulation of TAM in wheat seedlings. TAMs can be converted into serotonin (5-HT) or (and) IAA after *S. avenae* infestation and *F. graminearum* infection. Auxins may promote the rapid release of more plant nutrients; in addition, they may increase the opportunity for pathogen penetration and colonization, possibly because auxin can weaken the plant cell wall. Blue solid lines and light green lines represent the potential pathways of L-TRP catabolism. Green lines and arrows indicate the pathways for IAA signaling. Steel blue triangles depict the plant cell wall-degrading enzymes secreted by *F. graminearum*. Red question marks represent the pathways predicted from the literature. L-TRP, L- tryptophan; JA, jasmonic acid; IAA, indole-3-acetic acid; *TaCDC*, wheat tryptophan decarboxylases; ARF, auxin response factor; AUX/IAA, auxin/indole-3-acetic acid.

For a comprehensive understanding of auxin in the colonization strategies in plant-pathogen systems, we have focused on recent progress regarding the biosynthesis pathways for auxin. The essential amino acid L-TRP is the predominant building block for the biosynthesis of indole-3-acetic acid (IAA) in plants and microorganisms, such as *F. graminearum* (76–79). In addition, several studies have shown that *F. graminearum* inoculation leads to an increased biosynthesis of L-TRP and derived compounds in wheat and *Brachypodium distachyon* (80–82). The increase in L-TRP may then be used to stimulate the process of IAA biosynthesis in *F. graminearum*. However, the recent study showed that in liquid culture, *F. graminearum* rapidly metabolized exogenous L-TRP into indole tryptophol, no IAA was detected at any time point under the experimental conditions, and a series of genes associated with the function of L-TRP metabolism in the tricarboxylic acid cycle were identified (76). The exogenous application of JA to rice seedlings suggested that the JA accumulation in the plant tissue is important for converting L-TRP into tryptamine (TAM) using tryptophan decarboxylase (TDC) (83). Additionally, the latest study revealed that wheat plants of the susceptible cultivar Roblin exhibited higher *TaTDC* expression and then triggered the accumulation of TAM during *F. graminearum* infection (84). Together, in the context of the wheat–*F. graminearum* interactions under *S. avenae* exposure, the JA level in the environment produced after *S. avenae* infestation, and *F. graminearum* infection could trigger the conversion of L-TRP into TAMs (Figure 2). The experimental evidence acquired from wheat–*F. graminearum*, rice-*Nilaparvata lugens*, or the rice-*Sogatella furcifera* system revealed that TAM can be converted into serotonin by tryptamine 5-hydroxylase, which always involves one step (71, 85). Serotonin is also a regulator of plant growth and development, and rice mutants with inactivated CYP71A1 (exhibiting tryptamine 5-hydroxylase enzyme activity) created by CRISPR–Cas9 technology exhibited significantly increased salicylic acid levels and resistance to attackers when serotonin supplementation in an artificial diet enhances the fitness of herbivores (85). Similarly, serotonin has antagonistic interactions with the SA-mediated plant defense response, probably because serotonin and SA are derived from the same precursor. However, the significance of this pathway remains elusive in wheat–*F. graminearum* interactions under *S. avenae* influence. More work is required to unravel the significance and molecular mechanism of serotonin enhancing the performance of attackers.

In addition, the IAA biosynthesis pathways in fungi have been determined using external feeding experiments with L-TRP and its metabolized intermediates (74, 86). For instance, our recent studies demonstrated that when feeding with indolic intermediates, such as IPA, TAM, and IAN, *F. graminearum* could produce IAA (76, 77). TAM is one of the crucial intermediates that forms IAA. In comparison, the pathway of IAA synthesis derived from TAM is more complex, as it involves more possible intermediates (86, 87). According to the IAA biosynthesis pathways in *Arabidopsis*, most intermediates and enzymes predicted to participate in IAA biosynthesis have been identified in *F. graminearum via* comparative transcriptomic study. Approximately 135 candidate genes for the 14 enzymatic reactions were identified for part of the proposed pathways for IAA biosynthesis (88). In the TAM pathways, TAM can be converted into indole-3-acetaldehyde (IAAld) using monoamine oxidase enzymes and is then converted to IAA by indole-3-acetaldehyde oxidase (AAO). In addition, flavin monooxygenase-like enzymes can convert TAM to N-hydroxytryptamine (N-TAM) in a hydroxylation reaction, followed by the oxidation to form indole-3-acetaldoxime (IAOx). IAOx can undergo deamination to form IAAld or oxidation with cytochrome P450 (CYP) monooxygenase to form indole-3-acetonitrile (IAN) (89), followed by further conversion of IAN to form IAA by nitrilase enzymes, which catalyze the hydrolysis of the nitrile group. IAOx can also be formed from L-TRP in monooxygenation reactions (87). In addition, the biosynthesis of IAA is also initiated from the transamination of L-TRP by aminotransferase enzymes to form indole-3-pyruvate (IPA). The IPA pathway involves three steps, and its decarboxylation product IAAld is a key intermediate (74). This intermediate then shares the next step with the TAM pathway to convert IAAld to IAA. Thus, more work is required to unravel the IAA biosynthesis pathway(s) in *F. graminearum*.

## Potential Pathways of Auxin Accumulated in Wheat Plants and Roles Involved in Tripartite Interactions

The role of *Fusarium*-elicited IAA in microbe colonization has been determined in different crop plants; however, the experimental evidence that *F. graminearum*-elicited IAA stimulates its infection process is still required. The currently available knowledge on the mechanism of action of IAA in the fungal pathogen infection of *Arabidopsis* and other plant species can provide a baseline of information toward understanding the role of *F. graminearum*-elicited IAA in the tripartite interactions. There are three possible roles in which IAA can contribute to the promotion of the infection process.

First, *Fusarium*-elicited IAA, once distributed within the plant tissues, may lead to an elevated level of endogenous IAA during pathogen infection through the activation of host endogenous IAA production. The induction of IAA biosynthesis genes and the accumulation of IAA in infected tissues have been observed in *Arabidopsis* or rice (*Oryza sativa* L.) plants during infection with *F. oxysporum, M. oryzae*, or *Xanthomonas oryzae* (90–93). Our latest study suggested that most of the IAA production was likely derived from plant origin, as wheat plants exhibited higher IAA biosynthetic gene expression, while *F. graminearum* IAA biosynthetic genes were expressed at much lower levels during the infection. More experimental data supported these initial findings. It has been shown in rice that the exogenous treatment with IAA can induce the expression of some paralogs of the AAO and NIT families, which are important for auxin biosynthesis in plants (90). However, the pathway(s) associated with the IAA biosynthesis in wheat during *F. graminearum* infection have not yet been completely determined.

**Figure 3:**
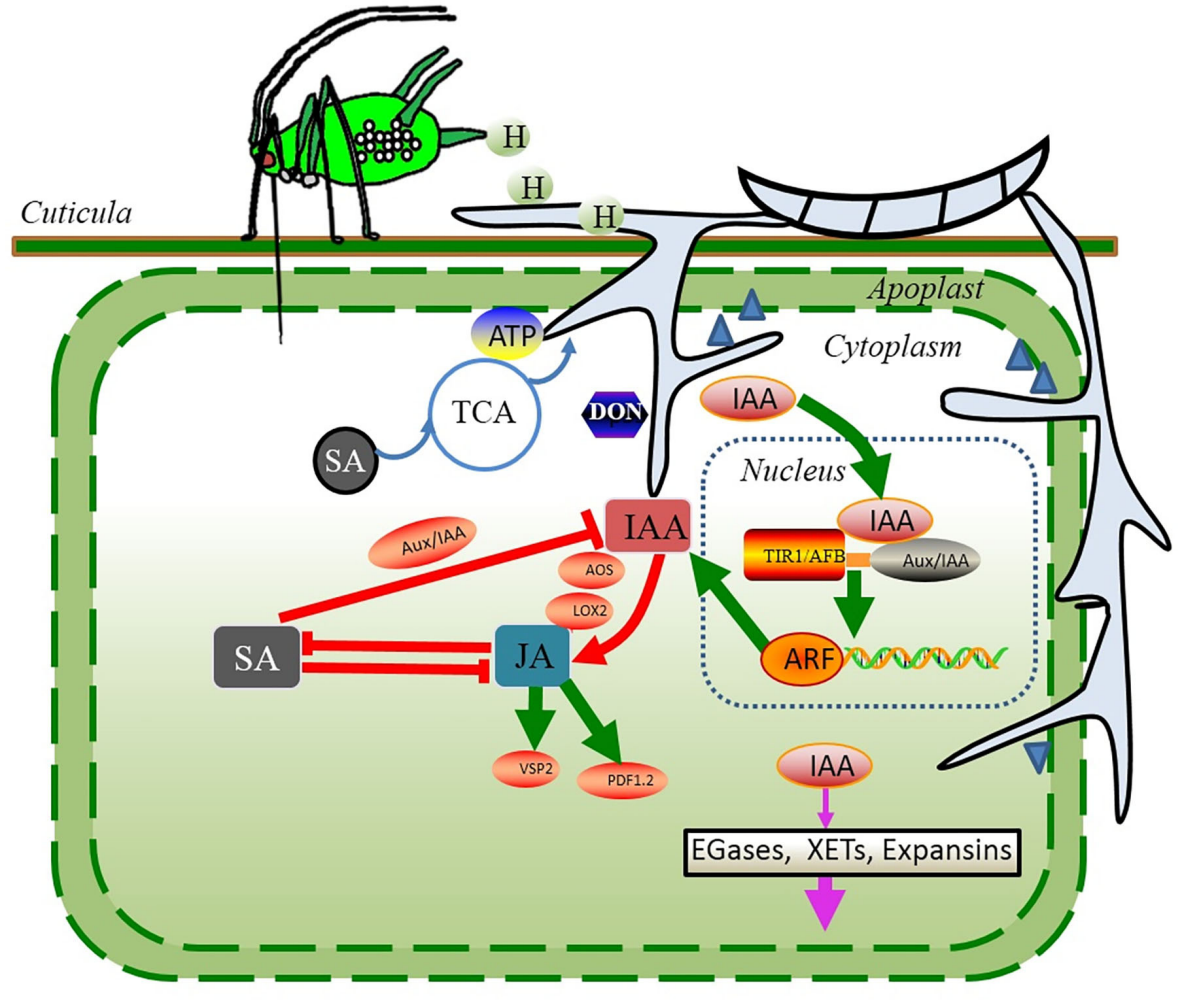
Potential strategies during the first few days of *F. graminearum* infection (biotrophic phase) in wheat continuously infested by *S. avenae*. *S. avenae* and their endosymbionts excrete a large amount of honeydew, which may provide ready-to-use carbon and nitrogen nutrients to accelerate the colonization of *F. graminearum*. To diminish the SA-dependent responses induced by aphid preinfestation, *F. graminearum* could metabolize exogenous SA as a source of carbon or manipulate host physiology to produce auxins that would fine-tune the host immune response. Auxins may attenuate the SA-dependent responses, possibly by activating the expression of JA biosynthesis-related genes and JA-regulated defense genes. Red solid lines represent the hormone-mediated upregulation (arrow) or inhibition (blunt-end line). Black blunt-end lines represent the inhibitory effects between the transcription factors. Moreover, *F. graminearum* significantly upregulates the expression of genes associated with the plant cell wall-degrading enzymes. Steel blue triangles depict the plant cell wall-degrading enzymes secreted by *F. graminearum*. Green lines and arrows indicate the pathways for IAA, JA, and SA signaling. Pink arrows indicate the pathway by which IAA weakens the plant cell wall. Blue lines and arrows indicate the pathways of SA degradation.

The responses associated with auxin accumulation can be key players in modulating the plant nutrient fluxes through auxin signaling (88). Auxin signaling (Figure 3) in plants involves the binding of auxin to coreceptors, proteasomal degradation of transcriptional repressors, release of transcriptional activators, and activation of auxin-dependent gene expression (94). Auxin signaling is initiated by the IAA perception: at high concentrations, auxin binds to auxin coreceptors, such as the Transport Inhibitor Response1/Auxin Signaling F-Box Proteins family (TIR1/AFBs) and Auxin/Indole-3-Acetic Acid (AUX/IAA) transcriptional repressors, forming TIR1/AFB–auxin–AUX/IAA complexes (94). The association of these components results in the proteasomal degradation of the repressor AUX/IAAs initiated by ubiquitin tagging and subsequent activation of the transcription factors, i.e., auxin response factor (ARF), to trigger the distinct auxin-dependent responses associated with stimulating cereal plants to grow and promote cereal susceptibility to pathogens (94).

**Figure 4:**
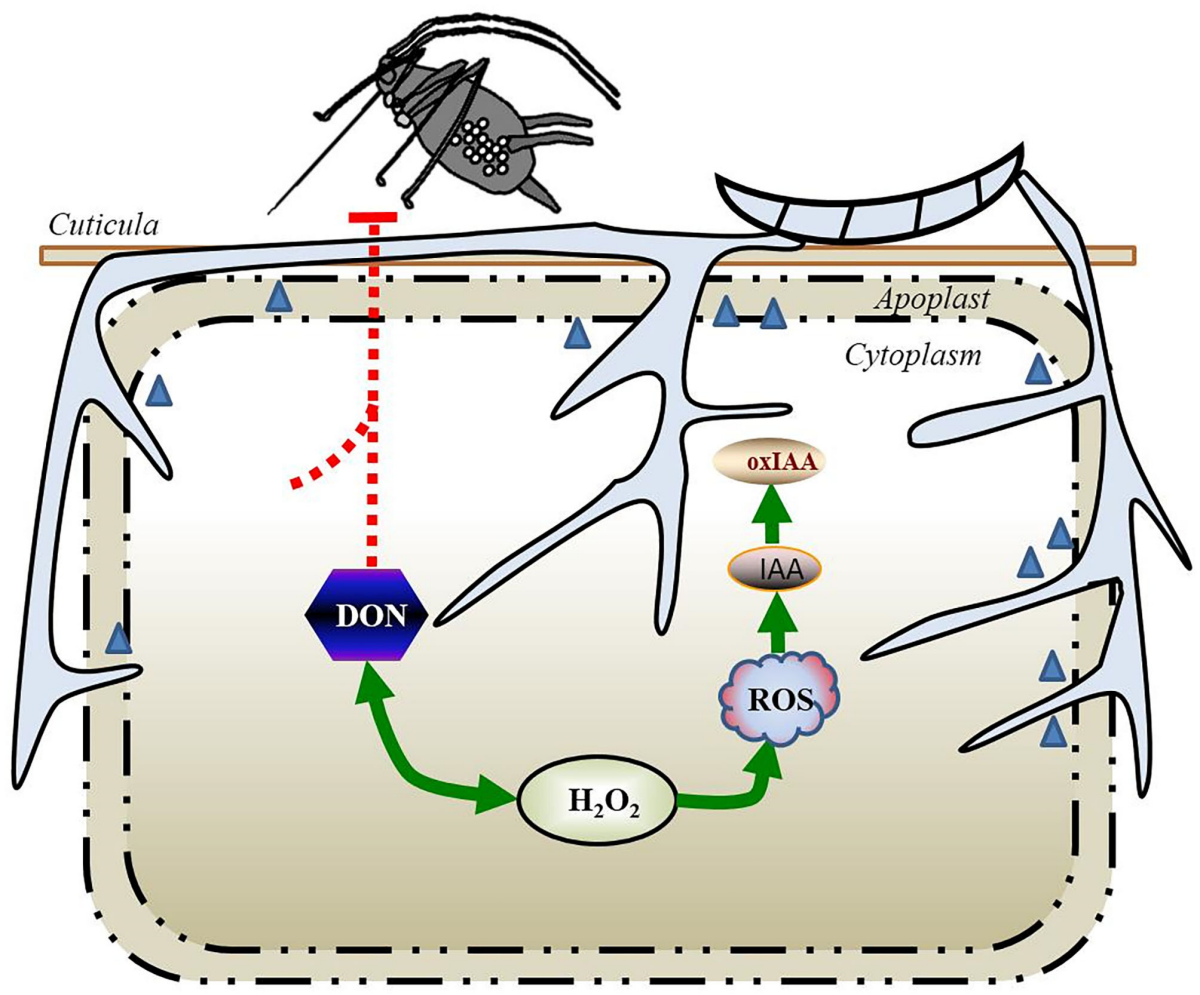
Model summarizing the tripartite interaction *S. avenae*–cereal–*F. graminearum* during the more invasive intracellular infection (necrotrophic phase) in wheat. The accumulation of DON becomes apparent and is necessary for the spread of the fungus in the rachis of wheat, and the whole spike becomes symptomatic, which results in a rapid decrease in the aphid populations. The rapid and transient generation of H_2_O_2_ induced by *S. avenae* and *F. graminearum* could significantly elevate the expression of the DON biosynthesis machine, while the DON production led to a further increase in ROS content. The extensive increase in the ROS content in host plants facilitates the oxidation of IAA to oxIAA and inactivates auxin-dependent responses. DON, deoxynivalenol; H_2_O_2_, hydrogen peroxide; IAA, indole-3-acetic acid; oxIAA, 2-oxindole-3-acetic acid; ROS, reactive oxygen species.

Second, IAA promotes rapid tissue growth, often associated with weakening of the plant cell wall during extension. Three types of cell wall structural proteins are involved in acid-induced wall extension, such as endo-β-1,4-glucanases (EGases), xyloglucan endotransglycosylases (XETs), and expansins. IAA can trigger the expression of expansins in many agricultural plant species that makes the plant cell wall more vulnerable to the pathogen invasion (91, 95). In the early phase of infection, *F. graminearum*-elicited IAA may stimulate the cell wall loosening and membrane leakage, thereby fueling the loss of water and nutrients. These nutrients would serve as an ideal supply to *F. graminearum* for the hyphal extension and mycotoxin production, accelerating the development of FHB disease symptoms (96). Supporting this possibility, ~134 candidate genes for the plant cell wall–degrading enzymes were significantly upregulated during the first few days of *F. graminearum* infection in wheat (97); these genes included genes predicted to encode enzymes catalyzing the cleavage of the main components of the plant cell wall, such as cellulose, hemicellulose, and pectin (Figures 3, 4).

Third, the extensive accumulation of IAA may attenuate the SA-dependent responses, possibly by activating the expression of JA biosynthesis-related genes and JA-regulated defense genes (Figures 2, 3), such as *AOS, LOX2* (lipoxygenase 2), and *VSP2* in plant seedlings (98). The extensive accumulation of JA in the host plants triggered the production of TAM, and TAM could be converted into IAA by *F. graminearum*. Many studies have shown that the IAA functions coordinately with other phytohormones to modulate the plant defense response (39, 70). The recent comparative transcriptomic study of wheat genotypes with different FHB resistance showed that the JA biosynthesis and response genes were strongly upregulated in the susceptible wheat cultivar Shaw after *F. graminearum* infection (99). In the same study, several members of the auxin response gene family *GH3s*, encoding hormone-amido synthetases, were also upregulated. *GH3s* can modulate the action of hormones. For instance, GH3.11 conjugates the amino acid isoleucine (Ile) to JA, forming JA-Ile conjugation, thereafter activating the JA-dependent signaling pathways (100). When it referred to the wheat–*F. graminearum* interactions under *S. avenae* influence, the phytohormones JA and IAA acted interdependently through the complex synergistic interactions to fine-tune their host defense responses.

Moreover, most of the reported GH3 proteins have IAA–amido synthetase activity, forming IAA-amino acid conjugations that contribute to the maintenance of auxin homeostasis and attenuate the IAA signaling. For instance, the overexpression of *GH3.8* or *GH3.2*, encoding IAA–amido synthetases, in transgenic rice plants led to diminished free IAA content and triggered broad-spectrum resistance to *M. oryzae* (90, 91). Under low auxin levels, AUX/IAA transcriptional repressors have been shown to heterodimerize with ARFs and repress their transcription (94). Reciprocally, increases in the SA levels upon herbivore infestation are associated with repression of auxin signaling through the transcriptional repression of auxin receptor genes (101). The repression of these auxin receptors, TIR1 and related F-box proteins would increase the stabilization of AUX/IAAs and attenuate the auxin signaling. Furthermore, recent evidence indicates that when IAA levels exceed the amount that plant cells can metabolize, excess IAA results in the localized accumulation of ROS (78). As discussed earlier, ROS accumulation, such as IAA- and SA-mediated ROS, would lead to a further increase in the DON production by *F. graminearum*, which subsequently enters into the phase of a rapid increase in the ROS content in the host plants (21).

Together, these results suggested that *Fusarium*-induced IAA could alter the plant physiology to increase its virulence, although large accumulation of IAA results in the cellular cytotoxicity to *F. graminearum* (77). The wheat plants or *F. graminearum* strains defective in IAA production or signaling will be required to clarify the exact role of IAA in the pathogenesis of *F. graminearum* and in the tripartite interaction *S. avenae*–wheat–*F. graminearum*.

## Concluding Remarks and Future Prospects

In the present review, we have gathered the available data on diverse factors known to affect the tripartite *S. avenae*–wheat–*F. graminearum* interaction that accelerates epidemics of FHB. Cereal aphid preinfestation has been shown to provide a large amount of ready-to-use nutrients *via* honeydew excretion that is associated with the accelerated growth and mycotoxin production by *F. graminearum*. The feeding behavior of aphid *S. avenae* also triggers the intricate and dynamic plant defense responses, such as rapid and transient generation of ROS. As ROS can stimulate the accumulation of mycotoxin DON, this plant defense response may play a key role in the favor of *F. graminearum* and accelerate infection. At the biotroph phase, especially in the first few days of infection, *F. graminearum* may have developed the ability to manipulate the plant hormonal balance for its own benefit by either producing or inducing production of the plant growth hormone auxin. A large amount of auxin in plants can have two potential consequences: (1) fast release of the plasma nutrients and increased opportunity for the successful penetration and colonization by pathogens and (2) interference with the SA-mediated signaling pathways by coordinating with the JA pathways to take advantage of these pathways for itself and aphids. Altogether, based on the present literature, the working hypothesis that aphid preinfestation accelerates the FHB disease progression from the aspects of environmental nutrients, auxin production, and hormone crosstalk, was synthesized at least in part by favoring the mycotoxin production.

This proposed working hypothesis also triggers important questions for future research and provides clues for elucidating this tripartite interaction. Although recent studies on the plant–pathogen interactions have identified auxin as a key player in the pathogenesis and plant defense, increasing evidence has pointed out that serotine also represses the SA-mediated defense responses. However, whether the preference pathway of biosynthesizing these two compounds is closely related to the types of attackers is unknown and further genetic study will be necessary to determine their mechanism in wheat-*F. graminearum* interaction under *S. avenae* influence. Moreover, the exact pathways and genes associated with the auxin are extensively accumulated in wheat plants during the first few days of infection by *F. graminearum*, which contribute to the knowledge required to comprehensively understand the role of FHB-induced IAA accumulation in repressing the host defenses or deploying *F. graminearum* virulence factors. Further work is necessary to develop *F. graminearum* mutants with the inactivated IAA biosynthesis genes in TAM-dependent pathways to determine their role in auxin accumulation in wheat plants. Thus, a combination of transcriptomics and metabonomics of hormone profiling in wheat-*F. graminearum* interaction under *S. avenae* influence will verify the working hypothesis synthesized in this review, which enables the development of integrated pest management measures to increase wheat production while maintaining food and feed quality.

## Author Contributions

KL and ZK conceived the study. KL, TO, and XW collected the data and led the writing of the manuscript. KL, ZK, TO, HZ, and XW participated in data interpretation and revised the manuscript. KL and TO prepared the figures. All authors have read and approved the manuscript for publication.

## Funding

This study was supported by the National 111 plan of China (program no. BP0719026), the Natural Science Basic Research Plan in Shaanxi Province of China (program no. 2019JCW-18), and the China Postdoctoral Science Foundation's funded project (2017M613228).

## Conflict of Interest

The authors declare that the research was conducted in the absence of any commercial or financial relationships that could be construed as a potential conflict of interest.

## Publisher's Note

All claims expressed in this article are solely those of the authors and do not necessarily represent those of their affiliated organizations, or those of the publisher, the editors and the reviewers. Any product that may be evaluated in this article, or claim that may be made by its manufacturer, is not guaranteed or endorsed by the publisher.
